# Smoking and *COX-2* Functional Polymorphisms Interact to Increase the Risk of Gastric Cardia Adenocarcinoma in Chinese Population

**DOI:** 10.1371/journal.pone.0021894

**Published:** 2011-07-14

**Authors:** Xue-Mei Zhang, Rong Zhong, Li Liu, Ying Wang, Ju-Xiang Yuan, Peng Wang, Chuang Sun, Zhi Zhang, Wen-Guang Song, Xiao-Ping Miao

**Affiliations:** 1 Department of Molecular Biology, College of Life Sciences, Hebei United University, Tangshan, China; 2 Ministry of Education Key Lab of Environment and Health, Department of Epidemiology and Biostatistics, School of Public Health, Tongji Medical College, Huazhong University of Science and Technology, Wuhan, China; 3 Department of Epidemiology, College of Public Health, Hebei United University, Tangshan, China; 4 Department of Oncology, Tangshan Gongren Hospital, Tangshan, China; Consiglio Nazionale delle Ricerche (CNR), Italy

## Abstract

**Background:**

Over-expression and increased activity of cyclooxygenase (COX)-2 induced by smoking has been implicated in the development of cancer. This study aimed to explore the interaction between smoking and functional polymorphisms of *COX-2* in modulation of gastric cardia adenocarcinoma (GCA) risk.

**Methods and Findings:**

Three *COX-2* polymorphisms, including –1195G>A (rs689466), –765G>C (rs20417), and 587Gly>Arg (rs3218625), were genotyped in 357 GCA patients and 985 controls. In the multivariate logistic regression analysis, we found that the –1195AA, –765GC, and 587Arg/Arg genotypes were associated with increased risk of GCA (OR = 1.50, 95% CI = 1.05–2.13; OR = 2.06, 95% CI = 1.29–3.29 and OR = 1.67, 95% CI = 1.04–2.66, respectively). Haplotype association analysis showed that compared with G_−1195_-G_−765_- G_Gly587Arg_, the A_−1195_-C_−765_-A_Gly587Arg_ conferred an increased risk of GCA (OR = 2.49, 95% CI = 1.54–4.01). Moreover, significant multiplicative interactions were observed between smoking and these three polymorphisms of –1195G>A, –765G>C, and 587Gly>Arg, even after correction by false discovery rate (FDR) method for multiple comparisons (FDR-*P*_interaction_ = 0.006, 5.239×10^−4^ and 0.017, respectively). Similarly, haplotypes incorporating these three polymorphisms also showed significant interaction with smoking in the development of GCA (*P* for multiplicative interaction = 2.65×10^−6^).

**Conclusion:**

These findings indicated that the functional polymorphisms of *COX-2*, in interaction with smoking, may play a substantial role in the development of GCA.

## Introduction

Gastric cardia adenocarcinoma (GCA) is the second leading cause of cancer-related mortality in the world, with more than 700,000 deaths annually [1], [2], [3]. In China, GCA is also one of prevalent fatal malignancies. During the past two decades, epidemiological studies have shown a steady decline in incidence of non-cardia gastric cancer but a continuously increased trend in incidence and mortality of GCA, thus emphasizing the importance of prevention strategy to GCA [4], [5]. Extensive studies have revealed several environmental factors involved in the development of GCA, including cigarette smoking, alcohol consumption, inflammation, and diet. Most significantly, smoking has been established by considerable studies as a causal factor for GCA [6], which was supported by a recent meta-analysis including 33 studies that smokers had 1.8 fold increased risk of GCA than never-smokers [7]. However, the underlying mechanism how smoking promotes GCA development remains to be fully elucidated. Recently, cumulative evidence has shown that smoking contributed to carcinogenesis potentially through induction of COX-2 and its downstream metabolites [8].

Cyclooxygenase-2 (COX-2), a key enzyme converting arachidonate to prostaglandins, was absent from normal cells unless rapidly induced by various carcinogens. For instance, the tobacco specified carcinogen, nicotine, has been shown to up-regulate COX-2 expression and activity *in vitro* and *in vivo* [9]. Moreover, in nicotine treated hamsters, COX-2 was significantly increased in gastrointestinal cancer [10], [11], [12]. Interestingly, in the gastric cancer cells with nicotine-induced COX-2-derived PGE2 release and cell proliferation, the COX-2 inhibitor SC-236 caused G1 arrest and abrogated nicotine-induced cell proliferation [13]. It is therefore concluded that COX-2 played key role in smoke-associated gastric cancer [8]. However, the expression level and activity of COX-2 induced by smoking may vary among individuals, and only small fraction of exposure individuals would develop to GCA during their life spans, suggesting genetic mechanism depending on COX-2 might be involved in susceptibility to smoke-associated GCA [14], [15].

Intriguingly, by direct sequencing and biochemical assays, we have previously identified three functional single nucleotide polymorphisms (SNPs) in *COX-2* gene, including –1195 G>A (rs689466) and –765G>C (rs20417) in promoter region and 587Gly>Arg (1759G>A, rs3218625) in coding region, of which, the G to A variant in –1195 locus created a c-myeloblastosis oncogene (c-MYB) binding site, resulting in higher transcriptional activity of *COX-2* [16]. The –765C allele might attribute to heighten smoking-induced expression of COX-2 by creating a binding site for phosphorylated NPM (P-NPM), which acted as a specific transcriptional inhibitor and was driven to cytoplasm once with smoking stimulation [17]. In addition, the 587Gly>Arg variant was associated with the enhanced activity of COX-2 *in vitro* by causing the Gly to Aly amino acid substitution in codon 587 of exon10 [18]. Therefore, an alternative hypothesis was motivated by solid biological plausibility that these three functional SNPs might interact with smoking to modulate the GCA risk. To test this hypothesis, –1195 G>A (rs689466), –765G>C (rs20417), and 587Gly>Arg (rs3218625) were analyzed in a case-control study consisting of 357 GCA cases and 985 controls in a Chinese Han population, and the interaction between these three SNPs and smoking exposure was investigated in modulation of GCA risk.

## Materials and Methods

### Study subjects

This study consisted of 357 GCA patients and 985 controls. All subjects were unrelated Han Chinese from Beijing city and its surrounding region. Patients were recruited between July 1999 and July 2005 at the Peking Union Hospital and Cancer Hospital, Chinese Academy of Medical Sciences (Beijing). The inclusion criteria for patients included histopathologically confirmed GCA, without previous chemotherapy or radiotherapy, and no restriction in regards to sex, age, or disease stage. The controls were cancer-free individuals randomly selected from a pool of 3000 normal individuals in the Beijing area during the same period. The selection criteria for controls included cancer-free individuals and frequency matching to cases by sex and age (±5 years). At recruitment, written informed consent was obtained from each subject, and the information on demographic characteristics, such as sex, age, and smoking status were collected via questionnaire. Subjects who had never smoked or smoked less than 1 cigarette per day and shorter than 1 year were defined as non-smokers; otherwise, they were considered as smokers (including current smokers and ex-smokers). This study was approved by the institutional review boards of the Chinese Academy of Medical Sciences Cancer Institute and Tongji Medical College of Huazhong University of Science and Technology.

### *COX-2* genotyping

Genomic DNA was extracted from whole blood samples of all subjects. Genotypes of three SNPs (including *COX-2* –1195G>A, –765G>C, and 587 Gly>Arg) were determined by polymerase chain reaction (PCR)-based restriction fragment length polymorphism (RFLP) methods as described previously [16]. Genotyping was performed without knowledge of the case or control status of the subjects. Genotypes determined by RFLP were further confirmed by direct sequencing in 30 random DNA samples, with the 100% concurrence rate of these two methods. Additionally, a 15% masked, random sample from cases and controls was tested twice by different investigators, and the results were concordant for all of the duplicate sets.

### Statistical analysis

χ^2^ test was used to compared the distribution of demographic characteristics between cases and controls. Hardy-Weinberg equilibrium for genotypes was tested by a goodness-of-fit χ^2^ test in control group. Multivariate logistic regression was used to evaluate the associations between GCA risk and smoking, *COX-2* genotypes or haplotypes. The interaction between smoking and *COX-2* SNPs were estimated via multiplicative interaction term and the stratified analysis of the effect of SNPs on GCA by smoking status. A two-tailed *P*<0.05 was used as the criterion of statistical significance. The final *P* values were adjusted by the false discovery rate (FDR) correction for multiple comparisons [19]. All statistical analyses were conducted by SPSS v13.0 software. Linkage disequilibrium (LD) of these three SNPs was analyzed using Haploview v4.0 [20]. Haplotypes composing these three SNPs were estimated using Phase v2.1 [21].

## Results

### Subjects characteristics

The demographic characteristics of all subjects are presented in Table 1. The cases and controls were matched well on sex and age distribution. There were 53.7% smokers among cases compared with 45% among controls. Significant difference in smoking status was observed between case and control groups (*P* = 0.006). In the logistic regression model, smokers had an increased risk of GCA compared with non-smokers after adjusting for sex and age (OR = 1.41, 95% CI = 1.08–1.84).

**Table 1:** Distributions of select characteristics among cases and controls.

Variable	Controls (*n* = 985)N (%)	Cases (*n* = 357)N (%)	*P* value
Gender			0.103
Male	810 (82.2)	307 (86.0)	
Female	175 (17.8)	50 (14.0)	
Age			0.453
≤45	102 (10.4)	29 (8.1)	
46–55	214 (21.7)	76 (21.3)	
56–65	380 (38.6)	134 (37.5)	
>65	289 (293)	118 (33.1)	
Smoking status			0.006
Nonsmoker	542 (55.0)	166 (46.5)	
Smoker	443 (45.0)	191 (53.5)	

### *COX-2* genotypes and GCA risk

The genotype distributions of the *COX-2* –1195G>A, –765G>C, and 587 Gly>Arg are shown in Table 2. All the genotypes of these three SNPs in controls conformed to Hardy-Weinberg equilibrium (*P* = 0.056, 0.463, and 0.394, respectively). For the –765G>C and 587 Gly>Arg polymorphsims, no variant homozygotes were observed in this study population. Frequencies for the variant alleles of −1195A, −765C, and 587Arg were 0.56, 0.09, and 0.08 in cases and 0.51, 0.05, 0.05 in controls, respectively.

**Table 2:** Genotype frequencies of *COX-2* SNPs and their association with GCA risk.

COX-2 genotypes	Cases/controls	OR (95% CI)†	FDR-*P* ‡
–1195G→A			
GG	69/217	Reference	
AG	175/527	1.03 (0.75−1.42)	0.847
AA	113/241	1.50 (1.05−2.13)	0.038
–765G→C			
GG	324/940	Reference	
GC	33/45	2.06 (1.29−3.29)	0.009
587Gly/Arg			
Gly/Gly	327/933	Reference	
Gly/Arg	30/52	1.67 (1.04−2.66)	0.033

†ORs and 95% CIs were calculated by unconditional logistic regression after adjusting for sex, age and smoking status.

‡*P* values were modified by the false discovery rate (FDR) correction for multiple comparisons.

In the logistic regression analysis, after adjusting for age, sex, and smoking status, individuals with the −1195AA genotype presented an elevated risk of GCA compared with those with the −1195GG genotype (OR = 1.50, 95% CI = 1.05–2.13). For the –765G/C SNP, individuals with the –765GC genotype had more than 2-fold increased risk of GCA (OR = 2.06, 95% CI = 1.29–3.29) compared with carriers of the –765GG genotype. Similarly, the 587Gly/Arg genotype also conferred 1.67 fold increased risk compared with the Gly/Gly genotype (95% CI = 1.04–2.66). Moreover, the effect of the −1195GG, –765GC, or 587Gly/Arg genotype achieved the significant level after correction by FDR for multiple comparisons (FDR-*P* = 0.038, 0.009, and 0.033).

### *COX-2* haplotypes and GCA risk

LD analysis showed that these three SNPs are not in discernible linkage disequilibrium in this study population (Table S1). As shown in Table 3, five common haplotypes composing these three SNPs were observed in this study population, of which, the A_−1195_-G_−765_-G_Gly587Arg_ haplotype was the most prevalent haplotype both in cases and controls (48.7% and 50.6%, respectively). Treating the G_−1195_-G_−765_-G_Gly587Arg_ haplotype as reference, the haplotype A_−1195_-C_−765_-G_Gly587Arg_ showed the significantly highest risk of GCA compare with other haplotypes (OR = 2.49, 95% CI = 1.54–4.01), and the G_−1195_-G_−765_-A_Gly587Arg_ was also significantly associated with the increased GCA risk (OR = 1.71, 95% CI = 1.01–2.88). Moreover, a significant allele-dose effect of haplotypes was observed in increasing risk of GCA (*P* for trend = 1.000×10^-5^).

**Table 3:** Distribution of *COX-2* haplotypes and their association with GCA.

Haplotype	Controls *n* = 985	Cases *n* = 357	OR (95%CI)†	*P* value
	No. of chromosomes (%)	No. of chromosomes (%)		
G_−1195_-G_−765_- G_Gly587Arg_A_−1290_-G_−1195_-G_−765_	913 (46.3)	290 (40.6)	Reference	
A_−1195_-G_−765_- G_Gly587Arg_	960 (48.7)	361 (50.6)	1.19 (0.10–1.43)	0.053
G_−1195_-G_−765_-A_Gly587Arg_	44 (2.2)	23 (3.2)	1.71 (1.01–2.88)	0.046
A_−1195_-C_−_765-G_Gly587Arg_	41 (2.1)	33 (4.6)	2.49 (1.54–4.01)	1.896×10^-4^
A_−1195_-G_−_765- A_Gly587Arg_	8 (0.4)	7 (1.0)	2.60 (0.93–7.23)	0.068

† ORs and 95% CIs were calculated by unconditional logistic regression after adjusting for sex, age and smoking status.

*P* for trend test = 1.000×10^−5^.

### Interaction of *COX-2* genotypes and smoking

In this current study, we evaluated the interaction of these three SNPs and smoking by stratified analysis and the multiplicative interaction term (Table 4). In the stratified analysis, none of the –1195G>A, –765G>C and 587Gly>Arg variants were associated with risk of GCA among nonsmokers, whereas smokers with the –1195GA+AA, –765GC or 587 Gly/Arg genotype all showed increased risk of GCA compared with non-smokers carrying the wild-type genotypes (OR = 1.64, 95% CI = 1.06−2.53; OR = 3.98, 95% CI = 2.06−7.69 and OR = 2.67, 95% CI = 1.36−5.23, respectively). Moreover, significantly multiplicative interaction were observed between these three *COX-2* SNPs and smoking, even after correction by FDR for multiple comparisons (FDR-*P*_interaction_ = 0.006, 5.239×10^-4^, 0.017).

**Table 4:** Stratify and interaction analysis between *COX-2* genotypes and Smoking status associated with the risk of GCA.

	−1195G>A				
Smoking status	GG		AG + AA		FDR*-P* ‡ for multiplicative interaction
	Cases/controls	OR (95% CI)†	Cases/controls	OR (95% CI)†	
nonsmoker	34/124	Reference	132/418	1.16 (0.75−1.77)	0.006
smoker	35/93	1.37 (0.78−2.38)	156/350	1.64 (1.06−2.53)	

Smoking status	−765G>C				
	GG		CG		FDR*-P* ‡ for multiplicative interaction
	Cases/controls	OR (95% CI)†	Cases/controls	OR (95% CI)†	
nonsmoker	155/515	Reference	11/27	1.31 (0.63−2.71)	5.239×10^-4^
smoker	169/425	1.32 (1.01−1.74)	22/18	3.98 (2.06−7.69)	

Smoking status	587 Gly/Arg	
	Gly/Gly		Gly/Arg		FDR*-P* ‡ for multiplicative interaction
	Cases/controls	OR (95% CI)†	Cases/controls	OR (95% CI)†	
nonsmoker	153/511	Reference	13/31	1.43 (0.73−2.81)	0.017
smoker	174/422	1.38 (1.05−1.81)	17/21	2.67 (1.36−5.23)	

† ORs and 95% CIs were calculated by unconditional logistic regression after adjusting for sex and age.

‡ *P* values for multiplicative interaction were adjusted by the false discovery rate correction for multiple comparisons.

### Interaction of *COX-2* haplotypes and smoking

We further explored the interaction of *COX-2* haplotypes and smoking in GCA (Table 5). Defining nonsmokers with the G_−1195_-G_−765_-G_Gly587Arg_ haplotype as the reference group, no significant association was observed between any haplotype and GCA risk among non-smokers, whereas smokes with the haplotype of G_−1195_-G_−765_-G_Gly587Arg_, A_−1195_-G_−765_-G_Gly587Arg_, G_−1195_-G_−765_-A_Gly587Arg_, A_−1195_-C_−765_-G_Gly587Arg_, or A_−1195_-G_−765_-A_Gly587Arg_ all showed elevated risk for developing GCA (OR = 1.38, 95% CI = 1.05–1.82; OR = 1.62, 95% CI = 1.24–2.11; OR = 2.20, 95% CI = 1.04–4.63; OR = 4.99, 95% CI = 2.54–9.81 and OR = 18.29, 95% CI = 2.11–158.24, respectively). Moreover, significantly multiplicative interactions were observed between *COX-2* haplotypes and smoking (*P*_interaction_ = 2.65×10^-6^).

**Table 5:** Stratify and interaction analysis between *COX-2* haplotypes and Smoking associated with the risk of GCA.

Haplotype	Nonsmoker		Smoker		*P* for multiplicative interaction
	Cases/controls	OR (95% CI)†; *P*	Cases/controls	OR (95% CI)†; *P*	
G_−1195_-G_−765_-G_Gly587Arg_A_−1290_-G_−1195_-G_−765_	136/502	Reference	154/411	1.38 (1.05–1.82); 0.021	2.650×10^-6^
A_−1195_-G_−765_-G_Gly587Arg_	172/524	1.22 (0.95–1.58); 0.124	189/436	1.62 (1.24–2.11); 3.928×10^-4^	
G_−1195_-G_−765_-A_Gly587Arg_	11/24	1.78 (0.85–3.74); 0.127	12/20	2.20 (1.04–4.63); 0.038	
A_−1195_-C_−765_-G_Gly587Arg_	11/25	1.58 (0.76–3.29); 0.226	22/16	4.99 (2.54–9.81); 3.240×10^-6^	
A_−1195_-G_−765_- A_Gly587Arg_	2/7	0.97 (0.20–4.75); 0.974	5/1	18.29 (2.11–158.24); 0.008	

† ORs and 95% CIs were calculated by unconditional logistic regression after adjusting for sex and age.

## Discussion

In this current study, we conducted a case-control study to investigate whether three functional polymorphisms in *COX-2*, including –1195G>A (rs689466), –765G>C (rs20417), and 587Gly>Arg (rs3218625), interacting with smoking, affect GCA risk in Chinese Han population. In stratified analysis, the effects of these three SNPs on GCA risk varied by smoking status. Furthermore, significant multiplicative interactions were observed between these three SNPs and smoking in GCA development. This study firstly suggested that the interaction of smoking with the *COX-2* –1195G>A, –765G>C, and 587Gly>Arg significantly contributed to GCA risk.

It was shown that COX-2 over-expression could increase proliferation, inhibit apoptosis, and enhance the invasiveness of cancer cells. Over-expression of COX-2 has been frequently seen in gastrointestinal malignancies, including pancreas, colon, non-cardia gastric cancer, and GCA [22], [23], [24], [25]. Interestingly, the smoking specified carcinogen, nicotine might act through β-adrenoceptors while NNK through both β-adrenoceptors and α7-nAChR, and induce COX-2 and its derived PGE2 in gastric cancer cells [13]. Cigarette smoke condensate could also induce COX-2 expression by activating nuclear factor kappa B (NF-κB) [26]. Furthermore, nicotine has been shown to facilitate gastric tumor angiogenesis and invasion, suggesting heighten expression of COX-2 induced by smoking might contribute to gastric carcinogenesis. There was evidence that SNPs altering COX-2 expression and activity have been implicated in risk of smoking-associated cancers, including lung, pancreatic, and non-cardia gastric cancer. However, few have done to investigate whether SNPs in *COX-2* affect GCA risk solely or in manner of interaction with smoking. Additionally, the effect of smoking on COX-2 enzyme was involved not only in the transcriptional expression but also in the enzymatic activity [13], [27], [28], [29]. Therefore, we analyzed three functional SNPs, –1195G>A and –765G>C in promoter region and 587Gly>Arg in coding region, in this current study.

The most significant finding in this study was that the variants of three functional SNPs identified by our previously studies interacted with smoking to increase risk of GCA. Of these three functional SNPs, –1195A-containing or –765C-containing *COX-2* promoter displays higher transcriptional activity, supported by examining the COX-2 expression level of these two variants by real-time PCR quantitation of *COX-2* mRNA in individual esophageal tissues [16]. Moreover, smoking exposure can rapidly induce COX-2 expression; hence, the joint effect of the variant and smoking has been expected to increase COX-2 expression. In this current study, significantly association between the –1195AA genotype and increased risk of GCA was only seen among smokers, and multiplicative interaction was observed between this variant and smoking, suggesting the cooperation of –1195G>A and smoking in modulation of GCA risk. Similar result was also observed for the other functional SNP of –765G>C in the promoter region, which was consistent with our previously finding that this SNP interacted with smoking to intensify the risk of pancreatic cancer. Moreover, the biochemical evidence that the markedly higher expression of COX-2 drove by the –765C-containing promoter than the –765G-containing promoter was only seen in the cells treated with smoke condensate has strongly supported this current result [17]. Additionally, the variant of 587Gly>Arg in coding region was also found to interact with smoking exposure to intensified GCA risk, being consistent with our previous finding that heavy smokers with the 587 Gly/Arg genotype presented the highest risk for ESCC compared with non-smokers with the wild-type genotype. Furthermore, biochemical evidence has suggested that the substitution of Gly to Arg, might affect COX-2 activity which was examined in the MCF-7 cells by enzymatic activity assays [18]. Considering that cigarette smoking also influenced COX-2 enzymatic activity, our result for the interaction of the 587 Gly>Arg variant with smoking was biologically plausible. Analysis was also performed on the haplotypes composing these three functional SNPs in GCA. The findings that the variant haplotypes was only associated with smokers' GCA risk and significant multiplicative interaction was observed between haplotypes and smoking further supported the hypothesis that the functional SNPs altering COX-2 expression and activity interacted with smoking to modulate GCA risk.

In summary, our study highlights the contribution of the interaction between smoking and functional SNPs in *COX-2* to GCA susceptibility, raising the prospect of research in personalized prevention strategies to smoking-associated GCA.

## Supporting Information

Table S1 Estimations of linkage disequilibrium (D'/r^2^) among the three SNPs of *COX-2* in this study population.
(DOC)

## References

[1] Vial M, Grande L, Pera M (2010). Epidemiology of adenocarcinoma of the esophagus, gastric cardia, and upper gastric third.. Recent Results Cancer Res.

[2] Jemal A, Siegel R, Xu J, Ward E (2010). Cancer statistics, 2010.. CA Cancer J Clin.

[3] Laheij RJ, Straatman H, Verbeek AL, Jansen JB (1999). Mortality trend from cancer of the gastric cardia in The Netherlands, 1969-1994.. Int J Epidemiol.

[4] Tran GD, Sun XD, Abnet CC, Fan JH, Dawsey SM (2005). Prospective study of risk factors for esophageal and gastric cancers in the Linxian general population trial cohort in China.. Int J Cancer.

[5] Li JY, Ershow AG, Chen ZJ, Wacholder S, Li GY (1989). A case-control study of cancer of the esophagus and gastric cardia in Linxian.. Int J Cancer.

[6] Savage SA, Abnet CC, Mark SD, Qiao YL, Dong ZW (2004). Variants of the IL8 and IL8RB genes and risk for gastric cardia adenocarcinoma and esophageal squamous cell carcinoma.. Cancer Epidemiol Biomarkers Prev.

[7] Tramacere I, La Vecchia C, Negri E (2011). Tobacco Smoking and Esophageal and Gastric Cardia Adenocarcinoma: A Meta-analysis.. Epidemiology.

[8] Huang RY, Chen GG (2011). Cigarette smoking, cyclooxygenase-2 pathway and cancer..

[9] de Souza Pereira R (2009). Selective cyclooxygenase-2 (COX-2) inhibitors used for preventing or regressing cancer.. Recent Pat Anticancer Drug Discov.

[10] Hein DW, Doll MA, Gray K, Rustan TD, Ferguson RJ (1993). Metabolic activation of N-hydroxy-2-aminofluorene and N-hydroxy-2-acetylaminofluorene by monomorphic N-acetyltransferase (NAT1) and polymorphic N-acetyltransferase (NAT2) in colon cytosols of Syrian hamsters congenic at the NAT2 locus.. Cancer Res.

[11] Schuller HM, Zhang L, Weddle DL, Castonguay A, Walker K (2002). The cyclooxygenase inhibitor ibuprofen and the FLAP inhibitor MK886 inhibit pancreatic carcinogenesis induced in hamsters by transplacental exposure to ethanol and the tobacco carcinogen NNK.. J Cancer Res Clin Oncol.

[12] Anto RJ, Mukhopadhyay A, Shishodia S, Gairola CG, Aggarwal BB (2002). Cigarette smoke condensate activates nuclear transcription factor-kappaB through phosphorylation and degradation of IkappaB(alpha): correlation with induction of cyclooxygenase-2.. Carcinogenesis.

[13] Shin VY, Jin HC, Ng EK, Yu J, Leung WK (2008). Nicotine and 4-(methylnitrosamino)-1-(3-pyridyl)-1-butanone induce cyclooxygenase-2 activity in human gastric cancer cells: Involvement of nicotinic acetylcholine receptor (nAChR) and beta-adrenergic receptor signaling pathways.. Toxicol Appl Pharmacol.

[14] Corley DA, Kubo A (2004). Influence of site classification on cancer incidence rates: an analysis of gastric cardia carcinomas.. J Natl Cancer Inst.

[15] Yang M, Guo Y, Zhang X, Miao X, Tan W (2007). Interaction of P53 Arg72Pro and MDM2 T309G polymorphisms and their associations with risk of gastric cardia cancer.. Carcinogenesis.

[16] Zhang X, Miao X, Tan W, Ning B, Liu Z (2005). Identification of functional genetic variants in cyclooxygenase-2 and their association with risk of esophageal cancer.. Gastroenterology.

[17] Zhao D, Xu D, Zhang X, Wang L, Tan W (2009). Interaction of cyclooxygenase-2 variants and smoking in pancreatic cancer: a possible role of nucleophosmin.. Gastroenterology.

[18] Zhao D, Zhang X, Guo Y, Tan W, Lin D (2009). Cyclooxygenase-2 Gly587Arg variant is associated with differential enzymatic activity and risk of esophageal squamous-cell carcinoma.. Mol Carcinog.

[19] Benjamini Y, Drai D, Elmer G, Kafkafi N, Golani I (2001). Controlling the false discovery rate in behavior genetics research.. Behav Brain Res.

[20] Barrett JC, Fry B, Maller J, Daly MJ (2005). Haploview: analysis and visualization of LD and haplotype maps.. Bioinformatics.

[21] Stephens M, Smith NJ, Donnelly P (2001). A new statistical method for haplotype reconstruction from population data.. Am J Hum Genet.

[22] Si J, Fu X, Behar J, Wands J, Beer DG (2007). NADPH oxidase NOX5-S mediates acid-induced cyclooxygenase-2 expression via activation of NF-kappaB in Barrett's esophageal adenocarcinoma cells.. J Biol Chem.

[23] Buskens CJ, Sivula A, van Rees BP, Haglund C, Offerhaus GJ (2003). Comparison of cyclooxygenase 2 expression in adenocarcinomas of the gastric cardia and distal oesophagus.. Gut.

[24] Sonoda R, Naomoto Y, Shirakawa Y, Fujiwara Y, Yamatsuji T (2010). Preferential up-regulation of heparanase and cyclooxygenase-2 in carcinogenesis of Barrett's oesophagus and intestinal-type gastric carcinoma.. Histopathology.

[25] Benamouzig R, Uzzan B, Martin A, Deyra J, Little J (2010). Cyclooxygenase-2 expression and recurrence of colorectal adenomas: effect of aspirin chemoprevention.. Gut.

[26] Duffield-Lillico AJ, Boyle JO, Zhou XK, Ghosh A, Butala GS (2009). Levels of prostaglandin E metabolite and leukotriene E(4) are increased in the urine of smokers: evidence that celecoxib shunts arachidonic acid into the 5-lipoxygenase pathway.. Cancer Prev Res (Phila).

[27] Huang RY, Chen GG (2011). Cigarette smoking, cyclooxygenase-2 pathway and cancer..

[28] Lin CC, Lee IT, Yang YL, Lee CW, Kou YR (2010). Induction of COX-2/PGE(2)/IL-6 is crucial for cigarette smoke extract-induced airway inflammation: Role of TLR4-dependent NADPH oxidase activation.. Free Radic Biol Med.

[29] Badawi AF, Habib SL, Mohammed MA, Abadi AA, Michael MS (2002). Influence of cigarette smoking on prostaglandin synthesis and cyclooxygenase-2 gene expression in human urinary bladder cancer.. Cancer Invest.

