# Supplementary material for: Smoking and COX-2 Functional Polymorphisms Interact to Increase the Risk of Gastric Cardia Adenocarcinoma in Chinese Population
Source: PLoS One. 2011 Jul 14;6(7):e21894. doi: 10.1371/journal.pone.0021894 (PMC3136492; doi:10.1371/journal.pone.0021894)
Supplement: Table S1 — Estimations of linkage disequilibrium (D'/r2) among the three SNPs of COX-2 in this study population. (DOC) [file pone.0021894.s001.doc]

Table S1. Estimations of linkage disequilibrium (D’/r^2^) among the three SNPs of *COX-2* in this study population.

|  | –1195G>A | –765G>C | 587 Gly>Arg |
| --- | --- | --- | --- |
| –1195G>A | – | 0.599/0.01 | 0.255/0.002 |
| –765G>C | – | – | 0.343/0.001 |
